# Psychosocial resources related to survival among non-robust community-dwelling older people: an 18-year follow-up study

**DOI:** 10.1007/s41999-020-00300-7

**Published:** 2020-03-05

**Authors:** Sirkku Lavonius, Marika Salminen, Tero Vahlberg, Raimo Isoaho, Sirkka-Liisa Kivelä, Maarit Wuorela, Minna Löppönen, Matti Viitanen, Laura Viikari

**Affiliations:** 1 Joint Authority for Päijät-Häme Health and Social Care, Elderly Care and Rehabilitation, Salpausselkä Rehabilitation Hospital, Tarjantie 78, 15950 Lahti, Finland; 2 Department of Geriatrics, Faculty of Medicine, Turku City Hospital, University of Turku, Kunnallissairaalantie 20, 20700 Turku, Finland; 3 City of Turku, Welfare Division, Yliopistonkatu 30, 20101 Turku, Finland; 4 Unit of Family Medicine, Faculty of Medicine, University of Turku, Lemminkäisenkatu 1, 20014 Turku, Finland; 5 Biostatistics, Institute of Clinical Medicine, University of Turku, Turku, Finland; 6 City of Vaasa, Social and Health Care, Ruutikellarintie 4, 65101 Vaasa, Finland; 7 Division of Social Pharmacy, Faculty of Pharmacy, University of Helsinki, 00014 Helsinki, Finland; 8 City of Raisio, Social and Health Care for Elderly, Sairaalakatu 5, 21200 Raisio, Finland; 9 Division of Clinical Geriatrics, NVS, Karolinska Institutet and Department of Geriatrics, Karolinska University Hospital, Huddinge, 14186 Stockholm, Sweden

**Keywords:** Frailty, Older people, Psychosocial, Resource, Survival

## Abstract

**Purpose:**

Psychosocial resources have been considered to be associated with survival among frail older adults but the evidence is scarce. The aim was to investigate whether psychosocial resources are related to survival among non-robust community-dwelling older people.

**Methods:**

This is a prospective study with 10- and 18-year follow-ups. Participants were 909 non-robust (according to Rockwood’s Frailty Index) older community-dwellers in Finland. Psychosocial resources were measured with living circumstances, education, satisfaction with friendship and life, visiting other people, being visited by other people, having someone to talk to, having someone who helps, self-rated health (SRH) and hopefulness about the future. To assess the association of psychosocial resources for survival, Cox regression analyses was used.

**Results:**

Visiting other people more often than once a week compared to that of less than once a week (hazard ratio 0.61 [95% confidence interval 0.44–0.85], *p* = 0.003 in 10-year follow-up; 0.77 [0.62–0.95], *p* = 0.014 in 18-year follow-up) and good SRH compared to poor SRH (0.65 [0.44–0.97], *p* = 0.032; 0.68 [0.52–0.90], *p* = 0.007, respectively) were associated with better survival in both follow-ups. Visiting other people once a week (compared to that of less than once a week) (0.77 [0.62–0.95], *p* = 0.014) was only associated with better 18-year survival.

**Conclusions:**

Psychosocial resources, such as regularly visiting other people and good self-rated health, seem to be associated with better survival among non-robust community-dwelling Finnish older people. This underlines the importance of focusing also on psychosocial well-being of frail older subjects to remain or promote their resilience.

## Key summary points

**Aim:**

To investigate whether psychosocial resources are associated with survival among non-robust community-dwelling older Finnish people during an 18-year follow-up.

**Findings:**

Psychosocial resources, such as good self-rated health and regularly visiting other people, were significantly associated with better survival of non-robust older people.

**Message:**

It is important to focus also on psychological well-being, together with physical activity and nutrition, of frail older people to remain or promoting their capacity.

## Introduction

Frailty is defined as a geriatric syndrome involving heightened vulnerability to stressors due to lowered physiological reserve and impairments in multiple systems. Frailty predicts adverse outcomes, such as falls, hospitalization, institutionalization and increased risk of death [1].

Psychosocial resources, contributors of resilience, have been considered to be related with the association between frailty and adverse outcomes [2, 3]. Psychological resources motivate healthy self-care behavior and promote resilience which does not only prevent frailty but also attenuate or reverse its negative effects [4, 5] by helping frail older adults to cope with stressful life events and maintain or initiate healthy behavior [5–7].

So far, the evidence of the moderating effect of psychosocial resources in the pathway from frailty to adverse outcome, such as mortality, is scarce and results vary between studies among community-dwelling older adults [8]. In the study of Op het Veld et al. [8], no moderating effects of resources, such as educational level, income, availability of informal care, living situation, sense of mastery and self-management abilities, were found on mortality among 2420 community-dwelling pre-frail and frail older Dutch people during the 2-year follow-up. In another Dutch study [9], 1665 mostly non-frail older adults were followed up for three years but no significant interaction effects between frailty and psychosocial resources (sense of mastery, self-efficacy, instrumental support and emotional support) for mortality were found. Follow-up periods of earlier studies have been short, and, therefore, studies with longer follow-ups are needed.

The aim of this study was to investigate whether psychosocial resources are related to survival among non-robust community-dwelling older Finnish people during an 18-year follow-up.

## Material and methods

### Study population

Subjects of this study were 1260 (82% of those invited) older adults (aged ≥ 64 years) living in the municipality of Lieto in Southwest Finland who participated in the longitudinal population-based epidemiological study, The Lieto Elderly Study, in 1998–99 [10]. Subjects living in institutional care (*n* = 65) or in sheltered housing (*n* = 18), with missing data of frailty (*n* = 51) or being robust (*n* = 217) were excluded from the analyses, leaving 909 non-robust (642 pre-frail and 267 frail) community-dwelling older subjects who were followed up for survival during an 18-year follow-up.

### Frailty

Frailty was assessed with Rockwood’s Frailty Index (FI) which consists of at least 30 deficits, such as symptoms, signs, and disabilities which are readily available in survey or clinical data [11]. Deficits of FI used in this study are seen in Table 1.

**Table 1:** Rockwood’s Frailty Index items

Needs help for toileting
Needs help for dressing and undressing
Needs help for preparing meals
Needs help for house work
Needs help for heavy household chores
Needs help for personal care
Needs help for moving about inside house
Arthritis or rheumatism
High blood pressure
Chronic bronchitis or emphysema
Diabetes mellitus
Heart disease
Cancer
Stomach or intestinal ulcers
Suffers from the effect of stroke
Urinary incontinence
Stool incontinence
Hip or femoral fracture
Shortness of breath
Angina pectoris
Other medical problems
No regular physical exercise
Vision problem
Hearing problem
Memory problem
Bodily pain
Speech problem
Resting tremor
Five or more medications
Difficulties carrying or lifting light loads
Mobility problem
Limited kind of amount of activity
Feeling tired all the time
Weight loss

### Psychosocial resources

Measures of psychosocial resources used in this study were living circumstances (1. living with someone, 2. living alone), education (1. at least basic education, less than basic education), satisfaction with friendships (1. satisfied, 2. rather satisfied, 3. rather disappointed or no friends), satisfaction with life (1. very satisfied, 2. satisfied, 3. somehow satisfied, disappointed or very disappointed), visiting other people (1. more than once a week, 2. once a week, 3. less than once a week), being visited by other people (1. more than once a week, 2. once a week, 3. less than once a week), having someone to talk to about anything (1. yes, 2. no), having someone who helps when needed (1. yes, 2. no), self-rated health (1. good, 2. moderate, 3. poor), and hopefulness about the future (1. often or always, 2. sometimes, seldom or never).

### Survival

Survival status of all participants until the end of December 2016 was obtained from the official Finnish Cause of Death Registry using unique personal identification numbers.

### Ethics

The study was conducted according to the guidelines of the Declaration of Helsinki. The Ethics Committee of the Hospital District of Southwest Finland approved the study protocol. Participants provided written informed consent for the study.

### Statistical analyses

Associations of age, gender, level of frailty and psychosocial variables with survival in 10- and 18-year follow-ups were examined with univariate Cox regression analysis. Proportional hazards assumptions were evaluated with martingale residuals. The follow-up periods were calculated from the baseline measurements to the date of death or to the end of the follow-up periods. Age-, gender- and level of frailty-adjusted multivariable Cox regression model included all psychosocial variables significantly (*p* < 0.05) associated with survival in univariate analyses. The adjusted interaction of gender and psychosocial variables as well as age and psychosocial variables was also tested to evaluate the modifying effect of gender and age on survival. No multicollinearity was found between explanatory variables (all correlations *r* < 0.40). The results were quantified by calculating hazard ratios (HR) with their 95% confidence intervals (95% CI). *p* values < 0.05 were considered statistically significant. All statistical analyses were performed using SAS System for Windows, version 9.4 (SAS Institute Inc., Cary, NC, USA).

## Results

The mean age of 909 non-robust community-dwelling participants was 73.2 (SD 6.4, range 64.0–97.0) years, and 541 (60%) were women. Baseline characteristics of the participants are seen in more detail in Table 2.

**Table 2:** Baseline characteristics of the among home-dwelling frail older adults (*n* = 909)

	*n* (%)
Age^a^	73.2 ± 6.4
Age	
64–69	320 (35)
70–74	253 (28)
75–79	177 (19)
≥ 80	159 (17)
Female	541 (60)
Living alone	282 (31)
Mini-mental state examination	
24–30	844 (93)
19–23	53 (6)
0–18	12 (1)
Body mass index	
< 25	258 (28)
25–29.9	399 (44)
30–24.9	188 (21)
≥ 35	61 (7)
Feelings of depression	78 (9)
Coronary heart disease^b^	414 (46)
Diabetes^c^	158 (17)
Having at least five medications in use	387 (43)
Ability to move outdoors independently	868 (95)
Ability to walk 400 m independently	819 (90)
Self-rated health	
Good	302 (33)
Moderate	456 (50)
Poor	151 (17)
Satisfaction with life	
Very satisfied	196 (22)
Satisfied	504 (56)
Somehow satisfied, disappointed or very disappointed	206 (23)

^a^Values are mean ± standard deviation

^b^ICD-10 codes I20–I25 and/or history of coronary by-pass surgery or coronary angioplasty and/or positive. Minnesota codes 1.1 or 1.2 as a sign of previous myocardial infarction in ECG and/or medication for coronary heart disease

^c^ICD-10 codes E10–E14 and/or serum fasting glucose > 7 mmol/l and/or treatment with anti-diabetic agents

### Survival

After the 10-year follow-up period, 63% of the participants, 57% of men and 67% of women, were alive. Corresponding proportions after the 18-year follow-up were 29%, 25% and 32%, respectively. The main causes of death were ischemic heart disease (ICD-10 codes I20–I25) (28%), cancer (C00–C97) (17%), dementia (F00–F03, G30) (16%), and cerebral insult (stroke [I63–I69, G45] 8%; hemorrhage [I60–I62] 3%) (11%).

### Psychosocial resources related to survival

In univariate analyses, age, gender, level of frailty and psychosocial factors such as living with someone, being satisfied or rather satisfied with friendships, being satisfied with life, visiting other people more than once a week or at least once a week, good or moderate self-rated health and hopefulness about the future were significantly associated with better survival both in 10- and 18-year follow-ups. Higher level of basic education was associated with better survival only in the 18-year follow-up (Table 3).

**Table 3:** Unadjusted hazard ratios (HR) and their 95% confidence intervals (95% CI) of psychosocial factors for mortality among home-dwelling frail older adults (*n* = 909) during a 10- and an 18-year follow-up

	10-year follow-up			18-year follow-up		
	HR	95% CI	*p* value	HR	95% CI	*p* value
Age			** < 0.001**			** < 0.001**
64–69 vs. 80+	**0.16**	**0.12–0.21**	** < 0.001**	**0.15**	**0.12–0.19**	** < 0.001**
70–74 vs. 80+	**0.23**	**0.17–0.31**	** < 0.001**	**0.27**	**0.21–0.33**	** < 0.001**
75–79 vs. 80+	**0.42**	**0.32–0.56**	** < 0.001**	**0.50**	**0.40–0.62**	** < 0.001**
Women vs. men	**0.68**	**0.55–0.84**	** < 0.001**	**0.76**	**0.65–0.89**	** < 0.001**
Pre-frail vs. frail	**0.31**	**0.25–0.38**	** < 0.001**	**0.34**	**0.29–0.40**	** < 0.001**
Living with someone vs. living alone	**0.69**	**0.55–0.86**	** < 0.001**	**0.69**	**0.59–0.81**	** < 0.001**
At least basic education vs. less than basic education	0.67	0.43–1.04	0.071	**0.61**	**0.45–0.84**	**0.002**
Satisfaction with friendships			**0.010**			**0.004**
Satisfied vs. rather disappointed or no friends	**0.59**	**0.40–0.87**	**0.008**	**0.66**	**0.50–0.86**	**0.002**
Rather satisfied vs. rather disappointed or no friends	**0.72**	**0.55–0.93**	**0.011**	**0.76**	**0.63–0.92**	**0.006**
Satisfaction with life			**0.002**			**0.040**
Very satisfied vs. somehow satisfied, disappointed or very disappointed	**0.55**	**0.40–0.77**	** < 0.001**	**0.78**	**0.62–0.98**	**0.029**
Satisfied vs. somehow satisfied, disappointed or very disappointed	**0.74**	**0.58–0.95**	**0.016**	**0.80**	**0.66–0.97**	**0.021**
Visiting other people			** < 0.001**			** < 0.001**
More often than once a week vs. less than once a week	**0.46**	**0.34–0.62**	** < 0.001**	**0.61**	**0.50–0.74**	** < 0.001**
Once a week vs. less than once a week	**0.55**	**0.42–0.73**	** < 0.001**	**0.59**	**0.48–0.72**	** < 0.001**
Being visited by other people			0.642			0.216
More often than once a week vs. less than once a week	0.89	0.69–1.15	0.373	0.86	0.71–1.03	0.095
Once a week vs. less than once a week	0.91	0.69–1.19	0.481	0.88	0.72–1.07	0.203
Having someone to talk to about anything vs. not having	0.97	0.74–1.26	0.790	1.05	0.86–1.27	0.650
Having someone who helps when needed vs. not having	0.96	0.66–1.39	0.814	1.09	0.82–1.46	0.538
Self-rated health			** < 0.001**			** < 0.001**
Good vs. poor	**0.32**	**0.23–0.43**	** < 0.001**	**0.39**	**0.32–0.49**	** < 0.001**
Moderate vs. poor	**0.49**	**0.38–0.63**	** < 0.001**	**0.49**	**0.40–0.60**	** < 0.001**
Hopefulness about the future often or always vs. sometimes, seldom or never	**0.65**	**0.52–0.82**	** < 0.001**	**0.73**	**0.61–0.86**	** < 0.001**

Factors significantly associated with survival are bolded

In multivariable analyses adjusted for age, gender and the level of frailty, visiting other people more than once a week (compared to that of less than once a week) and good self-rated health (compared to poor self-rated health) were associated with better survival both in 10- and 18-year follow-ups (Table 4). Visiting other people once a week (compared to that of less than once a week) was only associated with better 18-year survival.

**Table 4:** Adjusted hazard ratios (HR) and their 95% confidence intervals (95% CI) of psychosocial factors for mortality among home-dwelling frail older adults (*n* = 909) during a 10- and an 18-year follow-up

Factors significantly associated with survival in univariate analysis	10-year follow-up			18-year follow-up		
	HR	95% CI	*p* value	HR	95% CI	*p* value
Age			** < 0.001**			** < 0.001**
64–69 vs. 80 +	**0.21**	**0.15–0.30**	** < 0.001**	**0.19**	**0.15–0.25**	** < 0.001**
70–74 vs. 80 +	**0.28**	**0.20–0.39**	** < 0.001**	**0.32**	**0.26–0.41**	** < 0.001**
75–79 vs. 80 +	**0.50**	**0.37–0.68**	** < 0.001**	**0.59**	**0.47–0.75**	** < 0.001**
Women vs. men	**0.51**	**0.40–0.64**	** < 0.001**	**0.55**	**0.46–0.65**	** < 0.001**
Pre-frail vs. frail	**0.52**	**0.40–0.68**	** < 0.001**	**0.52**	**0.42–0.63**	** < 0.001**
Living with someone vs. living alone	0.83	0.65–1.08	0.168	0.85	0.71–1.03	0.092
At least basic education vs. less than basic education	1.13	0.75–1.71	0.563	0.91	0.65–1.26	0.560
Satisfaction with friendships			0.406			0.171
Satisfied vs. rather disappointed or no friends	1.13	0.75–1.71	0.563	0.96	0.72–1.28	0.762
Rather satisfied vs. rather disappointed or no friends	0.90	0.69–1.18	0.439	0.84	0.69–1.02	0.083
Satisfaction with life			0.998			0.121
Very satisfied vs. somehow satisfied, disappointed or very disappointed	1.00	0.67–1.47	0.986	1.30	0.98–1.70	0.065
Satisfied vs. somehow satisfied, disappointed or very disappointed	1.01	0.75–1.34	0.965	1.06	0.85–1.32	0.594
Visiting other people			**0.009**			**0.010**
More often than once a week vs. less than once a week	**0.61**	**0.44–0.85**	**0.003**	**0.77**	**0.62–0.95**	**0.014**
Once a week vs. less than once a week	0.79	0.59–1.07	0.125	**0.77**	**0.62–0.95**	**0.014**
Self-rated health			0.073			**0.027**
Good vs. poor	**0.65**	**0.44–0.97**	**0.032**	**0.68**	**0.52–0.90**	**0.007**
Moderate vs. poor	0.88	0.65–1.20	0.416	0.80	0.63–1.01	0.064
Hopefulness about the future often or always vs. sometimes, seldom or never	0.90	0.70–1.17	0.442	0.93	0.77–1.13	0.456

Factors significantly associated with survival are bolded

We also tested the interaction of gender and psychosocial resources as well as the interaction of age and psychosocial resources to evaluate the modifying effects of gender and age on survival (data not shown). The age- and the level of frailty-adjusted association of gender and psychosocial resources did not significantly differ between men and women either in 10- or 18-year follow-up. Significant interaction between age and basic education (adjusted with gender and the level of frailty) was found in 18-year follow-up (*p* = 0.038): higher education level predicted better survival in women (HR 0.62, 95% CI 0.40–0.97, *p* = 0.035) but not in men (0.77, 0.49–1.20, *p* = 0.245) during the 18-year follow-up.

## Discussion

In our study, not only lower age and lower level of frailty were most strongly related to better survival, but also psychosocial factors, such as living with someone, higher level of education, satisfaction with friendships, satisfaction with life, visiting other people regularly, good SRH and hopefulness about the future, were significantly related to better survival during the 10- and/or 18-year follow-ups in univariate analyses. In multivariable analyses adjusted with age, gender and the level of frailty, the association of good SRH remained significant during the 10-year follow-up; visiting other people at least on weekly basis remained significant both in 10- and 18-year follow-ups.

In earlier studies, psychosocial resources (e.g., educational level, availability of informal care, living situation, self-efficacy, instrumental support and emotional support) were neither found to moderate the effect of the level of frailty on mortality among pre-frail (77.8% of all frail subjects) and frail community-dwelling older adults during a 2-year follow-up [8] nor to buffer against mortality among community-dwelling older adults during a 3-year follow-up [9]. The inconsistency between the results of ours and those of earlier studies can probably be explained by a slightly different methodology and different psychosocial variables used and longer follow-up of our study. Also different frailty criteria were used; Fried’s phenotype model, defining frailty in physical terms, was used in the study of Op het Veld et al. [8], and cumulative deficit frailty index, a broader definition of frailty, in our study. In addition, participants in the study of Hoogendijk et al. [9] were younger than in our study and mostly non-frail. However, also in our study, majority (70.6%) of the frail participants were pre-frail as in the study of Op het Veld et al. [8].

In consistence with our results, several earlier studies have shown poor SRH being associated with higher mortality risk among general population of older adults [12–15]. In our study, also having a good SRH, compared to poor SRH, was associated with a 10-year survival but not with 18-year survival among frail older adults. Also according to the earlier studies, long-term predictive value of SRH for mortality has shown to be poorer than that of short-term among older adults [16, 17]. It has been argued that the validity of SRH is increased probably because individuals are including more objective information in their self-assessment of health [13]. On the other hand, older person defined as frail, based on objective measures, may not perceive his/herself as frail. In the study of Lucicesare et al. [18], poor SRH, measured using a 4-question SRH index, was an important predictor of mortality among robust older people but did not seem to increase mortality among frail older people.

Significant association of regularly visiting other people with survival in our adjusted multivariate analyses indicates the beneficial effects of social relationships on survival among frail older adults. In the English Longitudinal study of Ageing, lower levels of social relationships, e.g., loneliness and social isolation, were associated with premature mortality and with functional decline in older adults. Actually, the association of social relationships, e.g., loneliness, and frailty is bidirectional. Higher levels of frailty increase the likelihood of high levels of loneliness in the future [19]. Loneliness and lower level of social participation have shown to increase inactivity [20, 21] and physical frailty [19, 22] also in other studies. Although being socially active does not necessarily prevent or reduce feelings of loneliness [23], facilitating social connections could be beneficial for frail older adults. Unfortunately, according to a recent review, most interventions aimed to prevent or reduce frailty were focused on only one or two frailty markers, physical activity and/or nutrition, and seldom on psychological well-being [24]. Social factors, such as loneliness, are often challenging to modify. However, benefits have been reported from interventions promoting group activities and the use of existing public resources, such as libraries and volunteering groups, befriending, and teaching of IT skills to enable communication using internet-based activities [25]. Therefore, promotion of social, intellectual and emotional health and meaningful goals for life [26, 27] as well as a person-centered approach focusing remaining capacity [28, 29] and balancing factors for frailty [30], such as resilience [5], have been suggested for frail older adults.

The strengths of our study are the population-based sample of frail older adults, a reasonable sample size, and a long follow-up period enabling generalizability to the frail community-dwelling Finnish older population. Psychosocial measures used in this study are commonly used in Finnish population-based studies as well as in international studies. However, some limitations should be noted. First, in our study, no time-dependent covariates were used. Second, there may have been other important confounding factors, such as quality of life [27], socioeconomic status [5] and traumatic life experiences [27], which were unavailable in this study.

In conclusion, our findings suggest that psychosocial resources, visiting other people regularly and good SRH, are associated with survival among non-robust community-dwelling older people. Our findings underline the importance of focusing also on psychological well-being, together with physical activity and nutrition, of frail older people to remain or promoting their capacity.
